# Supplementary material for: Design rules for low-insertion-loss magnonic transducers
Source: Sci Rep. 2025 Mar 21;15:9806. doi: 10.1038/s41598-025-94474-4 (PMC11928728; doi:10.1038/s41598-025-94474-4)
Supplement: Supplementary file 1 — Supplementary Information 1. [file 41598_2025_94474_MOESM1_ESM.pdf]

1 Background information on micromagnetic simulations

1.1 Micromagnetic theory and modeling

Micromagnetics dynamics is intended to answer the question, how does the magnetization distribution $\mathbf{M}(\mathbf{r},t)$ changes in time under the influence of an effective magnetic field $\mathbf{H}_{\text{eff}}(\mathbf{r},t)$. This dynamics of $\mathbf{M}(\mathbf{r},t)$ is described by the Landau-Lifshitz equation :

$$\frac{\partial \mathbf{M}(\mathbf{r},t)}{\partial t} = -\gamma \mathbf{M}(\mathbf{r},t) \times \mathbf{H}_{\text{eff}}(\mathbf{r},t) - \frac{\alpha\gamma}{M_s}\left[\mathbf{M}(\mathbf{r},t) \times (\mathbf{M}(\mathbf{r},t) \times \mathbf{H}_{\text{eff}}(\mathbf{r},t))\right]. \tag{1}$$

Here γ is the gyromagnetic ratio ($\gamma = 2.210 \cdot 10^5 \mathrm{T}^{-1}\mathrm{s}^{-1}$), α is a damping constant (taking into account dissipation effects). The $\mathbf{H}_{\text{eff}}$ effective field is described as:

$$\mathbf{H}_{\text{eff}} = \mathbf{H}_{\text{exch}} + \mathbf{H}_{\text{Maxwell}} \quad . \tag{2}$$

Where $\mathbf{H}_{\text{Maxwell}}$ comes from the solution of Maxwell's equations and takes into account the external fields and the dipole-dipole interaction between the magnetic moments. $\mathbf{H}_{\text{exch}}$ models the exchange interactions between magnetic moments.

Solving the above equations is a daunting task and analytical solutions exist only for special cases. But the above equations are routinely solved numerically by publicly available software packages, such as OOMMF[1] and the mumax^3 code[2].

In the present paper we used mumax^3. mumax^3 employs a finite-difference discretization on a regular grid and leverages CUDA for parallel computations, allowing for efficient modeling of complex magnetic structures and dynamic phenomena such as spin waves.

Our numerical method is based on the combination of the above micromagnetic code mumax^3, a finite-element electromagnetic solver (FEMM)[3], and Matlab-based custom field-calculating routines. The magnetic field that originates from the waveguide and excites the film is calculated by FEMM am applied to the micromagnetic module, that determines magnetization dynamics. The magnetization dynamics gives rise to a time-dependent magnetic field, that induces voltage in the output waveguide. The Z-matrix-based circuit model is determined to describe the relation between the such-calculated input and output port currents / voltages.

In general, Eq. 1 describes a nonlinear dynamics. Naturally, the Z-matrix-based circuit model is valid only in the linear regime, when magnetization oscillations are small and this is what we assume throughout the paper.

1.2 Derivation of the spin-wave-wavelength

Small-amplitude wave-like solutions of Eq. 1 can be characterized by a dispersion relation, that describes the relation of the λ wavelength or k wavenumber to the oscillation frequency ω. This relation depends on the oscillation mode, the external field and the field geometry. In our work, determination of the λ wavelength is often important, since several design rules were determined in the keeping the λ constant and changing the bias field.

A reasonably accurate estimate of λ can be obtained from analytical dispersion relations, such as those derived by Kalinikos and Slavin[4], for a simple 2D film.

Alternatively, the value of the wavelength can be directly extracted from a snapshot of the micromagnetic simulations.

1.3 Derivation of the group velocity of the spin waves

The group velocity of spin waves is a crucial concept in the study of spin dynamics, representing the velocity at which the energy or information carried by the spin wave propagates through the material. Mathematically, the group velocity is defined as the derivative of the dispersion relation, which describes the relationship between the frequency (ω) and the wave vector (k) of the spin wave. Specifically, the group velocity (v_g) is given by:

$$v_g = \frac{d\omega}{dk} \tag{3}$$

For simple geometries, such as in ferromagnetic films or uniform bulk materials, the dispersion curve can often be derived analytically using established models, such as the one of Kalinikos and Slavin described above.

For more complicated geometries,the dispersion relation can be determined numerically. An efficient method (as described in e.g.[5]) is to apply a broadband excitation on the magnetic film, such as a spatially localized pulse: this creates excitations with a wide range of k numbers and ω frequencies. Fourier transforming $M_i(t,x)$ (where M_i is the oscillating component of the magnetization) gives $\mathbf{F}_{\mathbf{M_i}}(\omega,\mathbf{k_x})$, which is the dispersion relation.

References

1. Donahue, M. (1999), OOMMF User's Guide, Version 1.0, - 6376, National Institute of Standards and Technology, Gaithersburg, MD, [online], https://doi.org/10.6028/NIST.IR.6376 (Accessed February 3, 2025)
2. Vansteenkiste, A., Leliaert, J., Dvornik, M., Helsen, M., Garcia-Sanchez, F. & Waeyenberge, B. The design and verification of MuMax3. *AIP Advances*. **4** (2014,10), https://doi.org/10.1063/1.4899186
3. Meeker, D., *Finite Element Method Magnetics*, FEMM, https://www.femm.info, Accessed on January 19, 2024.
4. Kalinikos, B. & Slavin, A. Theory of dipole-exchange spin wave spectrum for ferromagnetic films with mixed exchange boundary conditions. *Journal Of Physics C: Solid State Physics*. **19**, 7013 (1986)
5. Venkat, Guru, D. Kumar, M. Franchin, O. Dmytriiev, Michał Mruczkiewicz, H. Fangohr, A. Barman, Maciej Krawczyk, and A. Prabhakar. "Proposal for a standard micromagnetic problem: Spin wave dispersion in a magnonic waveguide." IEEE Transactions on Magnetics 49, no. 1 (2012): 524-529.
