# Supplementary figures and images for: Design rules for low-insertion-loss magnonic transducers
Source: Sci Rep. 2025 Mar 21;15:9806. doi: 10.1038/s41598-025-94474-4 (PMC11928728; doi:10.1038/s41598-025-94474-4)

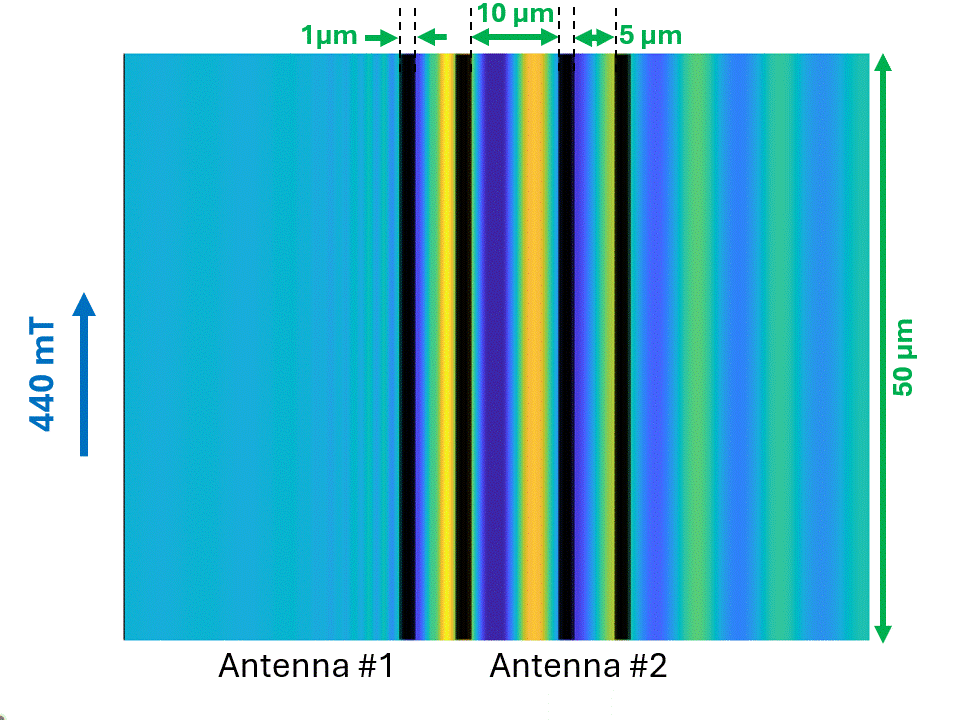

Supplement: Supplementary file 2 — Supplementary Information 2. [file 41598_2025_94474_MOESM2_ESM.gif]
